# Changes of lipid domains in *Bacillus subtilis* cells with disrupted cell wall peptidoglycan

**DOI:** 10.1111/j.1574-6968.2011.02417.x

**Published:** 2011-10-03

**Authors:** Katarína Muchová, Anthony J Wilkinson, Imrich Barák

**Affiliations:** 1 Slovak Academy of Sciences, Institute of Molecular BiologyBratislava, Slovakia; 2 Structural Biology Laboratory, Department of Chemistry, University of YorkYork, UK

**Keywords:** lipid domains, peptidoglycan, fluorescence microscopy, spheroplasts

## Abstract

The cell wall is responsible for cell integrity and the maintenance of cell shape in bacteria. The Gram-positive bacterial cell wall consists of a thick peptidoglycan layer located on the outside of the cytoplasmic membrane. Bacterial cell membranes, like eukaryotic cell membranes, are known to contain domains of specific lipid and protein composition. Recently, using the membrane-binding fluorescent dye FM4-64, helix-like lipid structures extending along the long axis of the cell and consisting of negatively charged phospholipids were detected in the rod-shaped bacterium *Bacillus subtilis*. It was also shown that the cardiolipin-specific dye, nonyl acridine orange (NAO), is preferentially distributed at the cell poles and in the septal regions in both *Escherichia coli* and *B. subtilis*. These results suggest that phosphatidylglycerol is the principal component of the observed spiral domains in *B. subtilis*. Here, using the fluorescent dyes FM4-64 and NAO, we examined whether these lipid domains are linked to the presence of cell wall peptidoglycan. We show that in protoplasted cells, devoid of the peptidoglycan layer, helix-like lipid structures are not preserved. Specific lipid domains are also missing in cells depleted of MurG, an enzyme involved in peptidoglycan synthesis, indicating a link between lipid domain formation and peptidoglycan synthesis.

## Introduction

The cell wall is essential for viability conferring structural integrity on bacterial cells. Its main function is to enable a high osmotic pressure inside the cell to be maintained. Despite its rigidity, the cell wall has to be porous enough to permit passage of specific solutes and adaptable enough to allow cell expansion (Bhavsar & Brown, 2006). The cell walls of eubacteria contain peptidoglycan (murein), a covalently linked macromolecular structure which is located immediately outside the cytoplasmic membrane and which is characteristic of these organisms (Rogers *et al*., 1980).

The cell walls of Gram-positive bacteria, such as *Bacillus subtilis*, are composed of a thick peptidoglycan layer to which anionic polymers, including cell wall teichoic acids, and specific proteins are covalently bound. Peptidoglycan is made of glycan strands cross-linked by short peptides to form a three-dimensional meshwork (Bhavsar & Brown, 2006). Although the initial steps of peptidoglycan and teichoic acid biosynthesis show some similarities, the sites of polymerization are distinct. Polymers of cell wall teichoic acids are formed intracellularly, whereas peptidoglycan polymerization occurs on the extracellular side of the membrane (Bhavsar & Brown, 2006).

The biosynthesis of peptidoglycan is a highly complex process that proceeds in several stages. Initially, in six cytoplasmic reactions catalyzed by MurA to MurF, the UDP-MurNAc-pentapeptide precursor, UDP-*N* acetylmuramic acid, is synthesized. MraY then catalyzes the reaction of this precursor with the membrane acceptor, undecaprenyl phosphate, to yield lipid I. The addition of *N*-acetylglucosamine (GlcNAc) to lipid I by MurG leads to lipid II, which represents the complete disaccharide-pentapeptide unit (van Heijenoort, 2001). Lipid II is then transferred to the outside of the membrane by lipid II flippase (Inoue *et al*., 2008). The next stage of peptidoglycan synthesis involves polymerization reactions on the outside surface of the cytoplasmic membrane catalyzed by penicillin-binding proteins (PBPs) and the incorporation of newly formed material into the existing peptidoglycan (Kawai *et al*., 2009). Peptidoglycan synthesis in rod-shaped *B. subtilis* cells takes place along the cylindrical part of the cell and at the cell septum, but not at the cell poles.

A direct link between peptidoglycan synthesis and the cytoskeletal system is suggested by the interactions between the glycosyltransferase MurG and the cytoskeletal protein MreB (Divakaruni *et al*., 2007; Mohammadi *et al*., 2007) as well as the direct association of several PBPs with MreB and the membrane proteins MreC and MreD (Leaver & Errington, 2005; van den Ent *et al*., 2006; Kawai *et al*., 2009).

The bacterial cytoplasmic membrane is an inhomogeneous highly dynamic structure, which contains distinct domains differing in their phospholipid and protein composition, giving rise to defined membrane microenvironments (Dowhan *et al*., 2004). It has been suggested that lipid domains containing anionic phospholipids play an important role in compartmentalization of specific proteins in the membrane (Epand & Epand, 2009). Recently, lipid microdomains similar to the lipid rafts described in eukaryotic cells have been discovered in *B. subtilis*. These lipid rafts, likely enriched in polyisoprenoids, can be isolated together with specific proteins such as homologues of eukaryotic Flotillin1 and other proteins involved in transport and signaling (Lopez & Kolter, 2010).

Organization of phospholipid molecules within the *B. subtilis* cytoplasmic membrane into specific domains enriched in phosphatidylethanolamine (Vanounou *et al*., 2003; Nishibori *et al*., 2005), cardiolipin (Kawai *et al*., 2004) and phosphatidylglycerol (Barák *et al*., 2008) has been observed. Phosphatidylethanolamine-rich domains are localized in the septal region of the membrane during vegetative growth and in the polar septum and forespore membrane during sporulation. Cardiolipin is preferentially localized at the cell poles and in the septal regions. Phosphatidylglycerol, the major negatively charged phospholipid in *B. subtilis*, was shown to be the principal component of lipid helix-like structures extending along the long axis of the cell. These lipid helices are recognized by the cell division protein MinD, indicating their possible involvement in cell division (Barák *et al*., 2008). These structures may also be employed in the localization of the secretory protein SecA, which exhibits a spiral pattern of localization dependent on the presence of anionic phospholipids (Campo *et al*., 2004). Moreover, it was shown recently that a cyclic lipopeptide antibiotic, daptomycin, preferentially interacts with lipid helices enriched in phosphatidylglycerol. Depletion of phosphatidylglycerol leads not only to decreased susceptibility to daptomycin but also to loss of the typical helical localization pattern observed in wild-type cells stained with a fluorescent derivate of daptomycin (Hachmann *et al*., 2009). Despite increasing recognition of their prevalence and importance in many cellular processes, it is still not understood how these domains of a unique phospholipid composition are formed.

Here, we present evidence that the presence of lipid domains in the cytoplasmic membrane is linked to the cell wall peptidoglycan. We analyzed lipid structures either in cells devoid of peptidoglycan through treatment with lysozyme or in cells depleted of MurG.

## Materials and methods

### Media and general methods

*Bacillus subtilis* cells were grown in LB media or DSM (Harwood & Cutting, 1990). Transformation of *B. subtilis* and other standard genetic techniques were carried out as described previously (Harwood & Cutting, 1990). When required, media were supplemented with erythromycin (1 μg mL^−1^) and lincomycin (25 μg mL^−1^). P_spac_-driven expression was induced using 1 mM isopropyl β-d-1-thiogalactopyranoside (IPTG).

### Bacterial strains

All *B. subtilis* strains used are derivatives of the wild-type PY79 strain (Youngman *et al*., 1984). Strain IB1302 (PY79 *murG::pMutin4erm*), which harbors an IPTG-inducible copy of *murG*, was created by the insertion of the plasmid pMmurG1 (*bla erm PspacΔmurG*) into the *murG* locus and mutants were selected for erythromycin/lincomycin resistance.

### Plasmids

Plasmid pMmurG1 was constructed using standard procedures and amplified in *E. coli* MM294 (*endA1 hsdR17 supE44 thi-1 recA*^*+*^) (Backman *et al*., 1976). A 688-bp DNA fragment of the *murG* gene was amplified from PY79 chromosomal DNA using the primers murGBS (5′-GATGATGATGGATCCGACTGGGGGAAAAAAGAAATGCGA-3′) and murGBE (5′-GATGATGATGGATCCCGGTGATATACAGCACTTGA TAG-3′). The PCR fragment was digested with BamHI (sites are underlined in the primer sequence) and cloned into similarly cleaved pMutin4, an integration vector used for gene inactivation (Vagner *et al*., 1998). Correct insertion in the vector was confirmed by restriction enzyme analysis and DNA sequencing.

### Preparation of the cell spheroplasts

*Bacillus subtilis* PY 79 strain was grown in liquid cultures in DSM. Each culture was inoculated from a fresh overnight plate to an OD_600 nm_ of 0.1 and grown to mid-exponential phase (OD_600 nm_ of 0.5). Samples of cell cultures were centrifuged (1 min at 2300 ***g***) and rapidly resuspended in 10 μL of 1× SMM (0.5 M sucrose, 20 mM maleic acid, and 20 mM MgCl_2_; pH 6.5) containing 1 mg mL^−1^ lysozyme (Errington, 1990; Ramamurthi & Losick, 2009). The mixture was mixed gently, and after approximately 10 min, a 0.5 μL sample was dropped directly onto 1% agarose pads prepared with LB medium. To check the viability of spheroplasts, staining with SYTO9 and propidium iodide from the Live/Dead BacLight Bacterial Viability and Counting Kit (Invitrogen) was performed.

### Fluorescence microscopy and image acquisition

*Bacillus subtilis* cultures were grown as liquid cultures in appropriate media as described above. To deplete MurG, the relevant culture was grown in DSM with 1 mM IPTG for 2 h and diluted into a medium lacking IPTG to an OD_600 nm_ of 0.05 and incubated for an additional 2 h. For membrane visualization, the fluorescent dye FM 4-64 (Molecular Probes) at concentrations of 0.2–1 μg mL^−1^ and nonyl acridine orange (NAO; Molecular Probes) at concentrations of 0.05–0.1 μg mL^−1^ were used. Samples were also stained with DAPI (0.2 μg mL^−1^) to visualize DNA. For imaging peptidoglycan biosynthesis, fluorescent vancomycin was used as described previously (Daniel & Errington, 2003; Tiyanont *et al*., 2006). Briefly, fluorescent vancomycin was prepared by mixing BodipyFL vancomycin (Molecular Probes) and unlabeled vancomycin in a 1 : 1 ratio. The vancomycin/BodipyFL vancomycin mixture was added to growing cultures to a final concentration of 1 μg mL^−1^. The culture was then incubated for a further 20 min before examination by microscopy. Cells were examined under the microscope on 1% agarose-covered slides. When it was necessary to increase the cell density, cells were concentrated by centrifugation (3 min at 2300 ***g***) and resuspended in a small volume of supernatant prior to examination by microscopy. All images were obtained with an Olympus BX61 microscope, equipped with an Olympus DP30BW camera. Olympus CellP imaging software or Olympus Image-Pro Plus 6.0 software was used for image acquisition and analysis. For image deconvolution and figure rendering, the Huygens Professional (Scientific Volume Imaging) software package was used.

## Results and discussion

### Helix-like lipid structures are not preserved in cells devoid of the peptidoglycan layer

In previous work using the fluorescent dyes FM 4-64, FM5-95, and FM1-43, we observed that the fluorescence signal in the membrane is distributed in a helical pattern. We also showed that phosphatidylglycerol is the main component of these helices (Barák *et al*., 2008). Analysis of strains in which DNA replication is inhibited revealed lipid domains identical to those observed in wild-type cells, indicating either that their formation is unaffected by inhibition of replication or that the structures already established are relatively stable (Muchová *et al*., 2010).

**Fig. 1:**
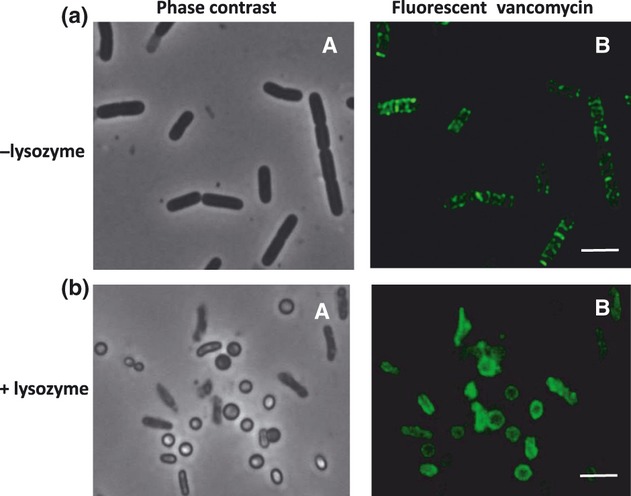
Staining of *Bacillus subtilis* PY79 cells with fluorescent vancomycin. Fluorescent vancomycin was prepared by mixing BodipyFL vancomycin (Molecular Probes) and unlabeled vancomycin in a 1 : 1 ratio. The mixture was added to the growing cultures to give a final concentration of 1 μg mL^−1^, and the culture was incubated for a further 20 min. (a) Fluorescent vancomycin staining of vegetatively growing cells. A helical pattern of the fluorophore was observed indicating a helical distribution of the peptidoglycan biosynthetic machinery. Phase-contrast (A) and fluorescence images (B). (b) Fluorescent vancomycin staining of cells treated with lysozyme. Staining was performed as described above. Cells were collected by centrifugation, resuspended in 1× SMM containing 1 mg mL^−1^ lysozyme, and further incubated for 10 min. The vancomycin fluorescence signal is localized around the circumference of the spheroplasts indicating the presence of residual peptidoglycan. There is no helical pattern to the observed fluorescence in these cells. Phase-contrast (A) and fluorescence images (B). Scale bars represent 2 μm.

Imaging of peptidoglycan biosynthesis with fluorescent derivatives of antibiotics demonstrated a helical distribution of the fluorescence signal along the cylindrical walls of *B. subtilis* cells (Fig. 1a) (Daniel & Errington, 2003; Tiyanont *et al*., 2006), a result suggesting that the peptidoglycan biosynthetic machinery is helically distributed. To examine whether the formation and persistence of helix-like lipid domains are dependent on cell wall peptidoglycan, we analyzed the membrane structure in cells in which the peptidoglycan layer has been disrupted by lysozyme treatment.

**Fig. 2:**
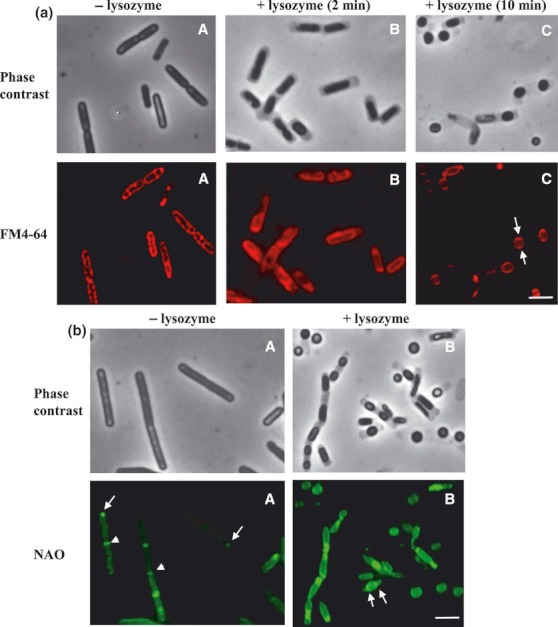
Lipid domains in spheroplasts of the wild-type *Bacillus subtilis* strain PY79. (a) Visualization of FM4-64 signals in spheroplasts. Spheroplasts were prepared by enzymatic removal of peptidoglycan with lysozyme. FM4-64-stained anionic phospholipids in wild-type cells resuspended in SMM buffer that were not treated with lysozyme (A) and in cells treated with lysozyme (B and C). (B) Cells with partially removed peptidoglycan after 2 min of lysozyme treatment, and (C) spheroplasts after 10 min of lysozyme treatment. While in the cells untreated with lysozyme, helix-like lipid structures stained with FM4-64 are visible, in spheroplasts, the FM4-64 signals are on the opposite sides of the cells. The arrows show the accumulation of anionic lipids stained with FM4-64. (b) Staining of spheroplasts with NAO to visualize cardiolipin domains. NAO-stained cardiolipin domains in wild-type cells that were not treated with lysozyme (A) and in cells treated with lysozyme (B). NAO fluorescence signals in spheroplasts preponderate at the former cell poles (arrows), indicating enrichment in cardiolipin at these sites as observed in vegetative cells. In cells not treated with lysozyme, arrows show its accumulation at the cell poles and arrowheads show its accumulation at the division sites. Scale bars represent 2 μm.

To prepare spheroplasts, we treated wild-type *B. subtilis* cells, grown as described in Materials and methods, with lysozyme. Enzymatic removal of the cell wall led to the formation of spherical cells that were stably and viably (as established by the Live/Dead assay) maintained in buffers containing a high concentration of sucrose (0.5 M). Having stained these cells with fluorescent vancomycin, residual cell wall peptidoglycan was detected, indicating partial removal by lysozyme (Fig. 1b). However, no helical structures were observable in these rounded lysozyme-treated cells in contrast to the untreated cells (Fig. 1a). The spherical cells were treated with FM4-64, which preferentially stains negatively charged phospholipids (Fig. 2aC). In these spheroplasts, we did not observe same lipid domains as detected in untreated cells (Fig. 2aA). This indicates that these structures are not preserved in cells devoid/depleted of peptidoglycan and/or that lysozyme treatment removes a determinant that contributes to the organization of lipids into helical structures.

It is known that a second anionic phospholipid present in *B. subtilis* membranes, cardiolipin, is distributed heterogeneously and forms specific cardiolipin-rich domains in the septal and polar membranes (Fig. 2bA) (Kawai *et al*., 2004). Treatment of spheroplasts with the cardiolipin-specific dye, NAO, revealed that the fluorescence signal is indeed focused at the former cell poles (Fig. 2Bb). The results show that while the spiral-like structures formed principally by phosphatidylglycerol and heralded by the FM4-64 dye are not preserved in protoplasted cells, the cardiolipin-rich domains are unchanged. Staining of spheroplasts with both fluorescence dyes revealed their co-localization in the most of protoplasted cells, indicating enrichment of these sites in the anionic phospholipids (not shown). It was reported recently that cardiolipin recognizes negative curvature of the lipid membrane in rod-shaped *E. coli* (Renner & Weibel, 2011). This finding predicts a more even distribution of cardiolipin in spheroplasts. The localization of cardiolipin, and possibly phosphatidylglycerol too, to specific sites in protoplasted *B. subtilis* cells may be caused by the presence of residual peptidoglycan after the lysozyme treatment.

The lysozyme treatment of cells leads to the partial removal of the peptidoglycan layer and the formation of rounded spheroplasts. The formation of lipid helix-like structures may depend on the presence of intact peptidoglycan. Alternatively, it may be determined by other factors associated with the maintenance of cell shape. Actin homologues determine cell shape in *B. subtilis*. The discovery that the cytoskeletal proteins Mbl, MreB, and MreBH are organized into helical structures (Jones *et al*., 2001; Defeu Soufo & Graumann, 2004) proximal to the intracellular surface of the cytoplasmic membrane led to speculation on the relationship between these internal structures and the peptidoglycan synthetic machinery. Moreover, staining of cells with a fluorescent derivative of vancomycin revealed that new peptidoglycan is incorporated into the cell wall in a helical Mbl-dependent manner (Daniel & Errington, 2003). Later, it was shown that the loss of the second major component of cell wall, wall teichoic acid polymers, has a dramatic effect on cellular morphology causing loss of rod shape (D'Elia *et al*., 2006).

However, other factors such as membrane curvature and/or preferential interactions between proteins and specific lipids may be determinants in the formation of lipid domains.

### Lipid domains are not preserved in cells depleted of the transferase MurG

**Fig. 3:**
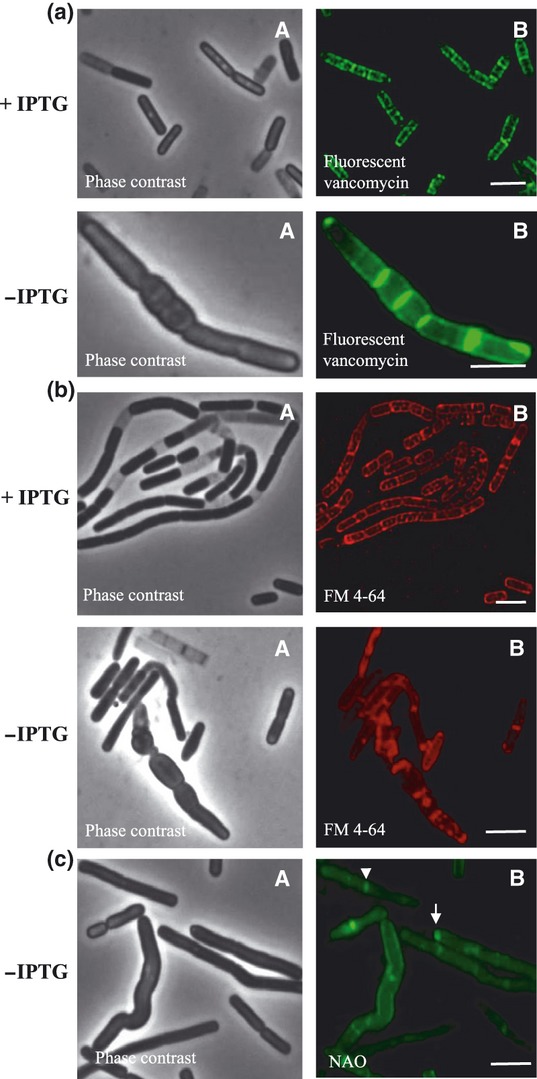
Peptidoglycan staining and localization of lipid domains in IB1302 cells depleted of MurG. Cell cultures were initially grown in the presence of 1 mM IPTG for 2 h before dilution to an OD_600 nm_ of 0.05 in media lacking IPTG and incubation for an additional 2 h. (a) Fluorescent vancomycin staining of peptidoglycan. In cells grown in the presence of IPTG, a helical pattern of fluorescence signal is observed, indicating a helical distribution of the peptidoglycan biosynthetic machinery similar to that in wild-type cells. However, in bulged cells that were grown without IPTG, no clear helical pattern of the fluorophore is observed. Instead, the fluorescence signal is accumulated at division sites where residual peptidoglycan synthesis occurred. Phase-contrast (A) and fluorescence images (B). (b) FM 4-64-stained anionic phospholipids. In the cells grown in the presence of IPTG, lipid domains similar to those observed in wild-type cells were detected. In cells, depleted of MurG, no such lipid structures are observable. Phase-contrast (A) and fluorescence images (B). (c) Cardiolipin domains stained with NAO. The arrow shows cardiolipin accumulation at the cell pole, and the arrowhead shows its accumulation at the division site in a way similar to that seen in wild-type cells. In bulged cells, the fluorescence signal is more evenly distributed in the membrane. Phase-contrast (A) and fluorescence images (B). Scale bars represent 2 μm.

The biosynthesis of peptidoglycan is a complex multistep process that has been investigated in various bacteria (van Heijenoort, 2001). The initial cytoplasmic steps involve the formation of the intermediates, lipid I and lipid II. Lipid II, which is the substrate for subsequent polymerization reactions, is formed by the action of the transferase MurG (van Heijennoort, 2007), a key enzyme in peptidoglycan biosynthesis. To study the effect of peptidoglycan depletion on the formation lipid domains, we prepared a strain in which an IPTG-inducible *murG* is introduced at the *murG* locus. Depletion of MurG by cell culture in the absence of IPTG leads to arrest of cell growth, cell bulging, and ultimately cell lysis (Fig. 3). Similar effects were observed after the depletion of another key enzyme of peptidoglycan synthesis, MurE (Leaver *et al*., 2009).

To determine whether, as expected, peptidoglycan biosynthesis is disturbed in cells depleted of MurG, we stained these cells with fluorescent vancomycin (Fig. 3a). In bulged cells, we observed a clear change in overall vancomycin staining and no helical distribution of the fluorescence signal. Instead, the signal is concentrated at division sites where a massive and rapid peptidoglycan synthesis occurs before the removal of inducer. Thus, the concentration of nascent peptidoglycan chains may remain high at these positions. It was shown previously that vancomycin binds to the terminal d-Ala-d-Ala of peptidoglycan precursors and to cell wall peptidoglycan that has yet to be cross-linked (Reynolds, 1989). As a result, labeled vancomycin can be used for staining sites of nascent peptidoglycan biosynthesis. A helical distribution of the vancomycin signal in wild-type *B. subtilis* cells implies a helical distribution of the sites of insertion of the nascent peptidoglycan into the cell wall (Daniel & Errington, 2003; Tiyanont *et al*., 2006). Accumulation of the fluorescence signal at division sites in cells depleted of either MurG or MurE (Daniel & Errington, 2003) shows that residual peptidoglycan synthesis occurs only at sites of septation.

To examine whether helix-like lipid structures are preserved under conditions of MurG depletion, we stained the *murG* mutant strain with FM4-64 (Fig. 3b). We observed that in the most of these cells, in bulged and also in rod-shaped cells, the FM dye does not exhibit a clear spiral pattern of localization, indicating that helix-like lipid domains are not preserved in cells with defects in peptidoglycan biosynthesis. To determine whether the distribution of cardiolipin-rich domains is also altered in these cells, we stained this mutant strain with NAO (Fig. 3c). Under conditions of MurG depletion, while in some more elongated cells, the fluorescence signal remains concentrated at the cell poles and division sites, in bulged cells, the signal is more evenly distributed in the membrane. These results suggest that disruption of peptidoglycan synthesis either prevents the formation and establishment of specific lipid domains such as those found in wild-type cells or causes disassembly of existing domains. It was shown previously that *E. coli* MurG binds to the membrane by direct interaction with phospholipids and preferential interaction with cardiolipin (van den Brink-van der Laan *et al*., 2003). Our results confirm this link between peptidoglycan synthesis and membrane lipid domains. A direct association of MurG and the cytoskeletal protein MreB in *E. coli* (Mohammadi *et al*., 2007) and the dependence of MurG localization on helical cables of MreB in *Caulobacter crescentus* (Divakaruni *et al*., 2007) also imply a close relationship between peptidoglycan synthesis and the preservation of cell shape.

The maintenance of cell shape and its connection with the peptidoglycan synthetic machinery has already been demonstrated (Leaver & Errington, 2005; Schimer & Errington, 2009; Mattei *et al*., 2010). Recently, coupled motion of MreB and the peptidoglycan elongation machinery around the cell has been observed (Dominquez-Escobar *et al*., 2011; Garner *et al*., 2011). It was shown that cell wall synthesis itself provides the driving force for MreB movement. Here, we present evidence that the formation and/or preservation of lipid domains within the cytoplasmic membrane may be linked to the integrity of cell wall peptidoglycan. We hypothesize that the association of the cytoskeletal system inside the cell with peptidoglycan synthesis outside the cell may place constraints on the distribution of phospholipids in the intervening membrane leading to the creation and stabilization of helix-like lipid domains. Further experiments are required to test this prediction, to fully understand how these domains are formed, and to determine what factors preserve these structures in the *B. subtilis* cell membrane.

## References

[b1] Backman K, Ptashne M, Gilbert AW (1976). Construction of plasmids carrying the cI gene of bacteriophage lambda. P Natl Acad Sci USA.

[b2] Barák I, Muchová K, Wilkinson AJ, O'Toole PJ, Pavlendová N (2008). Lipid spirals in *Bacillus subtilis* and their role in cell division. Mol Microbiol.

[b3] Bhavsar AP, Brown ED (2006). Cell wall assembly in *Bacillus subtilis*: how spirals and spaces challenge paradigms. Mol Microbiol.

[b34] van den Brink-van der Laan E, Boots J-WP, Spelbrink REJ, Kool GM, Breukink E, Killian JA, de Kruijff B (2003). Membrane interaction of the glycosyltransferase MurG: a special role for cardiolipin. J Bacteriol.

[b4] Campo N, Tjalsma H, Buist G (2004). Subcellular sites for bacterial protein export. Mol Microbiol.

[b5] D'Elia MA, Millar KE, Beceridge TJ, Brown ED (2006). Wall teichoic acid polymers are dispensable for cell viability in *Bacillus subtilis*. J Bacteriol.

[b6] Daniel RA, Errington J (2003). Control of cell morphogenesis in bacteria: two distinct ways to make a rod-shaped cell. Cell.

[b7] Defeu Soufo HJ, Graumann PL (2004). Dynamic movement of actin-like proteins within bacterial cells. EMBO Rep.

[b8] Divakaruni AV, Baida C, White CL, Goober JW (2007). The cell shape proteins MreB and MreC control cell morphogenesis by positioning cell wall synthetic complexes. Mol Microbiol.

[b9] Dominquez-Escobar J, Chastanet A, Crevenna AH, Fromion V, Weldich-Soldner R, Carballido-Lopez R (2011). Processive movement of MreB-associated cell wall biosynthetic complexes in bacteria. Science.

[b10] Dowhan W, Mileykovskaya E, Bogdanov M (2004). Diversity and versatility of lipid–protein interactions revealed by molecular genetic approaches. Biochim Biophys Acta.

[b35] van den Ent F, Leaver M, Bendezu F, Errington J, de Boer P, Lowe J (2006). Dimeric structure of the cell shape protein MreC and its functional implications. Mol Microbiol.

[b11] Epand RM, Epand RF (2009). Domains in bacterial membranes and the action of antimicrobial agents. Mol BioSyst.

[b12] Errington J, Harwood CR, Cutting SM (1990). Molecular biological methods for Bacillus.

[b13] Garner EC, Bernard R, Wang W, Zhuang X, Rudner DZ, Mitchison T (2011). Coupled, circumferential motions of the cell wall synthesis machinery and MreB filaments in *Bacillus subtilis*. Science.

[b14] Hachmann A-B, Angert ER, Helmann JD (2009). Genetic analysis of factors affecting susceptibility of *Bacillus subtilis* to daptomycin. Antimicrob Agents Chemother.

[b15] Harwood CR, Cutting SM (1990). Molecular Biological Methods for *Bacillus*.

[b36] van Heijennoort J (2007). Lipid intermediates in the biosynthesis of bacterial peptidoglycan. Microbiol Mol Biol Rev.

[b37] van Heijenoort J (2001). Formation of the glycan chains in the synthesis of bacterial peptidoglycan. Glycobiology.

[b16] Inoue A, Murata Y, Takahashi H, Tsuji N, Fujisaki S, Kato J (2008). Involvement of an essential gene, *mviN*, in murein synthesis in *Escherichia coli*. J Bacteriol.

[b17] Jones LJF, Carbalido-Lopez R, Errington J (2001). Control of cell shape in bacteria: helical, actin-like filaments in *Bacillus subtilis*. Cell.

[b18] Kawai F, Shoda M, Harashima R, Sadaie Y, Hara H, Matsumoto K (2004). Cardiolipin domains in *Bacillus subtilis* marburg membranes. J Bacteriol.

[b19] Kawai Y, Daniel RA, Errington J (2009). Regulation of cell wall morphogenesis in *Bacillus subtilis* by recruitment of PBP1 to the MreB helix. Mol Microbiol.

[b20] Leaver M, Errington J (2005). Roles for MreC and MreD proteins in helical growth of the cylindrical cell wall in *Bacillus subtilis*. Mol Mirobiol.

[b21] Leaver M, Dominguez-Cuevas P, Coxhead JM, Daniel RA, Errington J (2009). Life without a wall or division machine in *Bacillus subtilis*. Nature.

[b22] Lopez D, Kolter R (2010). Functional microdomains in bacterial membranes. Genes Dev.

[b23] Mattei P-J, Neves D, Dessen A (2010). Bridging cell wall biosynthesis and bacterial morphogenesis. Curr Opin Struct Biol.

[b24] Mohammadi T, Karczmarek A, Crouvoisier M, Bouhss A, Mengin-Lecreulx D, Blaauwen T (2007). The essential peptidoglycan glycosyltransferase MurG forms a complex with proteins involved in lateral envelope growth as well as with proteins involved in cell division in *Escherichia coli*. Mol Microbiol.

[b25] Muchová K, Jamroškovič J, Barák I (2010). Lipid domains in *Bacillus subtilis* anucleate cells. Res Microbiol.

[b26] Nishibori A, Kusaka J, Hara H, Umeda M, Matsumoto K (2005). Phosphatidylethanolamine domains and localization of phospholipids synthases in *Bacillus subtilis* membranes. J Bacteriol.

[b27] Ramamurthi KS, Losick R (2009). Negative membrane curvature as a cue for subcellular localization of bacterial protein. P Natl Acad Sci USA.

[b28] Renner LD, Weibel DB (2011). Cardiolipin microdomains localize to negatively curved regions of *Escherichia coli* membranes. P Natl Acad Sci USA.

[b29] Reynolds PE (1989). Structure, biochemistry and mechanism of action of glycopeptide antibiotics. Eur J Clin Microbiol Infect Dis.

[b30] Rogers HJ, Perkins HR, Ward JB (1980). Microbial Cell Walls and Membranes.

[b31] Schimer K, Errington J (2009). Influence of heterologous MreB proteins on cell morphology of *Bacillus subtilis*. Microbiology.

[b32] Tiyanont K, Doan T, Lazarus MB, Fang X, Rudner DZ, Walker S (2006). Imaging peptidoglycan synthesis in *Bacillus subtilis* with fluorescent antibiotics. P Natl Acad Sci USA.

[b33] Vagner V, Dervyn E, Ehrlich SD (1998). A vector for systematic gene inactivation in *Bacillus subtilis*. Microbiology.

[b38] Vanounou S, Parola AH, Fishov I (2003). Phosphatidylethanolamine and phosphatidylglycerol are segregated into different domains in bacterial membrane. A study with pyrene-labelled phospholipids. Mol Microbiol.

[b39] Youngman P, Perkins JB, Losick R (1984). Construction of a cloning site near one end of Tn917 into which foreign DNA may be inserted without affecting transposition in *Bacillus subtilis* or expression of the transposon-borne *erm* gene. Plasmid.

